# The cell surface-associated rhamnose-glucose polysaccharide represents the receptor of *Streptococcus thermophilus* bacteriophage P738

**DOI:** 10.1128/aem.01238-25

**Published:** 2025-09-12

**Authors:** Zoe Kampff, Irina Sadovskaya, Evgeny Vinogradov, Brian McDonnell, Douwe van Sinderen, Jennifer Mahony

**Affiliations:** 1 School of Microbiology, APC Microbiome Ireland, University College Cork 8795, Cork, Ireland; 2 UMR 1158 BioEcoAgro, Institut Charles Viollette, Univ. Littoral Côte d'Opale, USC ANSES, INRAe, Univ. Artois, Univ. Lille, Univ. Picardie Jules Verne, Univ. Liège, Junia 27023, Boulogne‑sur‑Mer, France; 3 Institute for Biological Sciences, National Research Council Canada 6356, Ottawa, Ontario, Canada; Centers for Disease Control and Prevention, Atlanta, Georgia, USA

**Keywords:** receptor, saccharides, functional genome analysis, cell wall, starter culture, fermentation

## Abstract

*Streptococcus thermophilus* is a key lactic acid bacterial species extensively used in dairy fermentations, where bacteriophage (phage) infections pose a significant threat to production efficiency. While the cell surface receptors for four of the five currently known streptococcal phage genera have previously been identified, the specific host-encoded receptor for the P738 phage group has not been elucidated to date. In this study, P738 bacteriophage-insensitive mutants of *S. thermophilus* UCCSt50 were isolated, and whole-genome sequencing revealed mutations in a glycosyltransferase-encoding gene within the *rgp* locus, responsible for the biosynthesis of the cell wall-associated rhamnose-glucose polysaccharide (RGP). Compositional and methylation analysis demonstrated that loss of a terminal rhamnose residue from the RGP side chain underpinned the observed resistance to P738 binding and infection. Fluorescent binding assays validated the biological functionality of the predicted receptor binding protein of P738, confirming the critical role of the RGP side chain in phage adsorption. These findings reveal a strict requirement for a complete tetrasaccharide side chain structure for P738 binding, contrasting with the less stringent requirements observed for the *Brussowvirus* SW13. This work offers valuable insights to guide the development of phage-robust starter cultures through knowledge-based strain selection and rotation strategies, thereby supporting more sustainable and reliable dairy fermentation processes.

**IMPORTANCE:**

*Streptococcus thermophilus* is one of the most extensively applied members of the lactic acid bacteria, being extensively used as a bacterial starter culture in the production of fermented foods, including cheeses and yogurt. Bacteriophage infections present a significant threat to industrial dairy fermentations, which can lead to substandard production processes and product quality. Understanding phage-host interactions, which commence with the recognition and binding of a given bacteriophage to a particular host-encoded, cell surface-associated receptor, is essential for developing strategies to minimize phage-related risks in dairy fermentations. This study identifies the specific cell surface polysaccharide structure produced by *Streptococcus thermophilus* as the receptor required for infection by phage P738, bridging a key knowledge gap in phage-host interactions in this important industrial bacterial species. By elucidating how phage P738 recognizes and binds to its host, this work offers valuable information to aid strain selection and the development of phage-robust starter cultures.

## INTRODUCTION

*Streptococcus thermophilus* is a member of the lactic acid bacteria (LAB) that has long been associated with the production of fermented dairy products (1). Based on its long history of safe use in food fermentations and human consumption, *S. thermophilus* is the only streptococcal species to be granted “generally recognized as safe” status by the Food and Drug Administration and “qualified presumption of safety” status by the European Food Safety Authority (2). *S. thermophilus* is commercially one of the most valuable LAB globally, as strains of this species are used extensively in bacterial starter cultures in the production of fermented foods. From an applied perspective, certain metabolic products of *S. thermophilus,* particularly exopolysaccharides (EPSs), contribute to desirable textures, mouthfeel, and flavor of yogurts and hard cheeses (3, 4).

One of the most significant and consistent threats to these fermentation processes is infection of starter bacteria by bacterial viruses, commonly called bacteriophages (or phages). Phage infection of strains within starter cultures may disrupt growth and milk acidification rates, leading to slower or inconsistent production processes or, in severe cases, failed fermentations (5). The persistence of phages in the dairy industry negatively impacts production efficiency and product quality with serious economic consequences. Historically, two groups of dairy streptococcal phages were identified based on their mode of DNA packaging, as well as the number of major structural proteins (6). These groups are now termed the Moineauviruses (originally termed the *cos* phages) and Brussowviruses (originally termed the *pac* phages) (7). Subsequently, three additional and genetically distinct phage groups were identified, termed the *Vansinderenvirus* (8), *Piorkowskivirus* (formerly termed the 987 group) (9, 10), and P738 (11) groups. Phages belonging to the *Vansinderenvirus* and P738 genera exhibit sequence similarity to phages of pathogenic and non-dairy streptococci, while genomes representative of *Piorkowskivirus* group phages appear to be hybrids of dairy streptococcal and lactococcal phage genome modules (12, 13).

Considerable research efforts have been invested in defining the mechanisms by which *S. thermophilus* responds to the challenge of phage infection. Most notable among these are studies pertaining to clustered regularly interspaced short palindromic repeats (CRISPR) systems in *S. thermophilus* (14–17). The CRISPR-Cas systems in *S. thermophilus* provide adaptive immunity against bacteriophages by incorporating short DNA segments from invading phages into the CRISPR spacer arrays, enabling the bacterium to recognize and defend against subsequent infections (16). Beyond these systems, receptor modification has been shown to play a crucial role in phage resistance, in addition to other strategies (18, 19). The host-encoded receptors for a handful of dairy streptococcal phages have been identified as saccharidic moieties, which are presented on the surface of the host cell as tightly or loosely cell-associated structures and which correspond to cell wall rhamnose-glucose polysaccharides (RGPs) and exopolysaccharides, respectively (20–23). Biosynthesis of these complex glycans is performed by an enzymatic machinery encoded by two distinct loci termed the *rgp* and *eps* gene clusters, respectively, which are located in the chromosomes of *S. thermophilus* strains. Members of the *Piorkowskivirus* phage group recognize an EPS receptor (20, 21), while the RGP is essential for *Brussowvirus* infection (23). Furthermore, a potential link has been proposed between *Moineauvirus* phylogeny and host *eps* genotypes, implicating EPS as a possible receptor for members of this group, though this has not been experimentally validated to date (22). Similarly, the EPS may serve as the receptor for members of the *Vansinderenvirus* phage group, based on host range data and receptor binding protein (RBP) analyses (12, 24, 25). Collectively, these findings emphasize the essential role of *S. thermophilus* cell wall polysaccharides (CWPSs) in mediating phage interactions. Isolation of non-CRISPR-mediated bacteriophage-insensitive mutants (BIMs) has been instrumental in identifying host-encoded phage receptors, further informing the selection and development of robust, phage-resistant *S. thermophilus* strains for industrial applications (26).

As mentioned above, the *rgp* locus encodes the biosynthetic machinery for RGP production. The RGP molecule of a given *S. thermophilus* strain is composed of two distinct saccharidic components: a conserved rhamnan-rich backbone and a variable side chain. The backbone structure is believed to be embedded in and covalently attached to the peptidoglycan layer, while the side chain structure is covalently linked to the backbone structure and exposed at the cell surface, where it may become the specific “docking” point for phages with a cognate receptor binding protein (27). The variable side chain may differ in subunit composition, length, and it is this unique composition and structure that dictate the highly specific interactions of certain dairy streptococcal phages and their hosts. This has been proven experimentally, where the side chain structure is required for host binding by a member of the *Brussowvirus* phage group (23). Synthesis of the rhamnan backbone structure commences with the transfer of a GlcNAc moiety from UDP-GlcNAc to Und-P to form the precursor Und-P-P-GlcNAc, which is mediated by the RgpG/TagO equivalent encoded by a glycosyltransferase encoded beyond the *rgp* gene cluster (28). Subsequently, RgpA transfers the first rhamnose residue to the Und-P-P-GlcNAc foundation, and the extension of the backbone structure is a function of the rhamnosyltransferases/glycosyltransferases encoded within the 3′ end of the *rgp* gene cluster. The rhamnan subunits are transferred to the outer face of the cell membrane through the activity of the ABC-transport proteins RgpC/D. The side chain structure is synthesized independently and initiated by the transfer of GlcNAc/GalNAc moiety to the lipid carrier Und-P by the priming glycosyltransferase. The glycosyltransferases encoded within the 5′ region of the *rgp* gene cluster contribute to the extension of the side chain subunit structure, and the subunits are transferred to the outer face of the membrane by a flippase also encoded in this region of the cluster. Hierarchical clustering analysis of the *rgp* gene clusters of 78 *S*. *thermophilus* strains revealed considerable genetic diversity of *rgp* loci at the strain level, enabling the identification of seven distinct *rgp* genotypes (termed *rgp*1–7). Additionally, *S. thermophilus* strains can be classified based on the gene content associated with the biosynthesis of the rhamnan backbone (Bt) and variable side chain (Vt) (28). A detailed analysis of the rhamnan backbone and side chain-associated regions of *rgp* loci has so far revealed three distinct backbone genotypes (Bt1-3) and five variable side chain genotypes (Vt1-5), each corresponding to distinct RGP chemical structures (28).

To date, the receptors recognized by members of four of the five genera of dairy streptococcal phages have been identified. Currently, only two members of the recently described P738 genus have been isolated and characterized, namely P738 and D4446, which were isolated in Germany and Senegal, respectively (11). Despite the relatively recent identification of these phages, their isolation from two distant geographical locations and high level of sequence relatedness suggest that these phages are likely to be present in other factories and may remain undetected due to the current lack of tools to identify them. It is also likely that these phages will increase in abundance if suitable host strains continue to be applied in industrial fermentations. Therefore, in the current study, we sought to identify the specific host-encoded receptor of the P738 group through analysis of bacteriophage-insensitive mutants of the host strain of phage P738. Whole-genome sequencing and single nucleotide polymorphism (SNP) analysis of two such BIMs were applied to identify the genes responsible for the observed resistance. Additionally, the structure of the RGP moiety of a representative mutant was determined and compared to the parent strain to establish the impact of the mutation on the host cell wall RGP structure. Finally, the biological functionality of the predicted RBP of P738 was validated through fluorescent binding assays, while these assays also confirmed that the adsorption step of the phage cycle was impaired in the generated BIMs. This study provides the first molecular-level insights into P738 phage-host interactions, characterizing the genetic and structural basis of phage receptor binding.

## RESULTS

### P738-insensitive derivatives of *S. thermophilus* UCCSt50 are non-CRISPR mediated

The host-encoded receptors of members of four of the five known genera of dairy streptococcal phages have been defined to date (20–23). In the present study, we aimed to identify the receptor for the fifth genus of dairy streptococcal phages, i.e., the P738 genus (11). Following exposure of the host strain, *S. thermophilus* UCCSt50, to phage P738 (10^8^ PFU/mL, multiplicity of infection ~0.5–1), a panel of 20 individual survivors that were presumed to be BIMs was colony-purified for further characterization. Presumptive P738-resistant BIMs were generated at a frequency of ~10^−5^. All isolates selected for analysis retained an incomplete or complete phage-resistance phenotype after colony purification and overnight incubation in LM17 broth. Full resistance of a BIM was defined as the complete absence of plaques following exposure to phage P738 (at a level of 10^8^ PFU/mL, obtained for parent strain UCCSt50), while incomplete resistance was characterized by a significant reduction in phage susceptibility, where BIMs exhibited more than a five-log reduction in phage titer compared to the wild-type strain (Table S1). To validate the integrity of the BIMs as derivatives of *S. thermophilus* UCCSt50, *rgp* genotyping using the recently described dual multiplex PCR system was performed on each isolate as well as the parent strain. Furthermore, the CRISPR1 and CRISPR3 loci and spacer arrays of the isolates were also amplified and compared to those of the parent strain. In all cases, the PCR-generated DNA fragments were shown to be identical in size to those of the parent strain. Among the panel of 20 strains, two BIMS named F1B and F2A were selected for whole-genome sequencing to determine the underlying genetic alterations that mediate the observed P738 phage resistance.

To investigate potential CRISPR-mediated resistance mechanisms, the genomes of UCCSt50 and the selected BIMs were sequenced to completion with whole-genome sequencing performed using long read sequencing technology paired with Illumina short-read sequencing (a summary of the general genome characteristics is presented in Table S2). To establish if CRISPR spacer acquisitions underpinned the observed resistance in one or both strains, the genome sequences of BIMs F1B and F2A were analyzed using CRISPRCasFinder (https://crisprcas.i2bc.paris-saclay.fr/CrisprCasFinder/Index) to compare the composition of their spacer arrays against that of the parent strain and were observed to be identical (Table S3).

### P738 phage resistance is mediated by a mutation in a gene within the *rgp* cluster

To establish which genes were responsible for the observed P738-resistant phenotype, SNP analysis was performed. This revealed distinct single point mutations in a gene within the *rgp* gene cluster for both BIMs, while F2A also exhibited an additional mutation (Table 1).

**TABLE 1:** Summary of SNPs identified in derived BIMs of *S. thermophilus* UCCSt50

BIM	SNP position	Base modification	Amino acid changes	*orf* no.	Predicted function
F1B	1,344,528	g → a	Trp^269^ → stop	*orf*07020	Glycosyltransferase
	1,371,556	c → a	Ser^28^ → stop	*orf*07170	DMT family transporter
F2A	1,230,230	c → t	Pro^52^ → Ser^52^	*orf*06475	UDP-glucose-4-epimerase
	1,345,056	a → c	Asp^93^ → Ala^93^	*orf*07020	Glycosyltransferase

Both BIMs were found to harbor a distinct mutation within *orf*07020, a gene encoding a predicted glycosyltransferase within the *rgp* cluster of *S. thermophilus* UCCSt50, which is presumed to be involved in RGP side chain biosynthesis (28). This gene corresponds to *orf*06960 in a previous genome annotation and is located immediately upstream of *orf*06955, which has been shown to be essential for a *Brussowvirus*-type phage infection (23). In the case of F1B, the incorporated mutation introduces a stop codon in place of a tryptophan (Trp269) residue, resulting in the truncation of the encoded protein (269 aa instead of 322 aa). For the derivative F2A, the mutation within *orf*07020 culminates in an amino acid substitution (aspartic acid to alanine). Additionally, F1B harbors a mutation in *orf*07170, a gene predicted to encode a DMT family transporter. F2A also contains a mutation in *orf*06475, which is annotated as *galE*, and predicted to encode a UDP-glucose-4-epimerase, which is involved in the interconversion of UDP-galactose to UDP-glucose. Given that *orf*07020 was mutated in both BIMs and since low concentrations of UDP-Glucose and UDP-Galactose are associated with exopolysaccharide production in *Lactococcus* (29)*,* the functional role of this gene in mediating P738 phage resistance was also evaluated.

To establish if the identified mutations within *orf*07020_UCCSt50_ were responsible for the acquired P738 phage resistance phenotype observed in the BIMs, the native *orf*07020_UCCSt50_ was cloned into the high copy expression vector pNZ44 (30) and introduced into BIM F1B and F2A (designated F1B::pNZ44-07020_UCCSt50_ and F2A::pNZ44-07020_UCCSt50_, respectively; Table 2). The introduction of the native gene resulted in the restoration of phage sensitivity to the same order of magnitude as that of UCCSt50 for F2A (efficiency of plaquing [EOP] = 0.9 ± 0.1) and F1B (EOP = 0.35 ± 0.33) (Table 3), confirming the role of *orf*07020_UCCSt50_ in the P738 infection process. Furthermore, F1B harboring the control plasmid (F1B::pNZ44) and a plasmid-cured derivative (F1B::pNZ44-07020C_UCCSt50_) exhibited complete phage resistance (Table 3). To evaluate the possible role of *galE* in the observed phage resistance of BIM F2A, the native *orf*06475_UCCSt50_ was cloned and transformed into BIM F2A, but its presence was not associated with restoration of phage sensitivity, indicating that it does not contribute to P738 phage resistance. These findings reinforce the involvement of *orf*07020_UCCSt50_ in P738 phage resistance in both BIMs.

**TABLE 2:** Bacterial strains, bacteriophages, plasmids, and primers used in this study

Strains			
	Description		Source
*S. thermophilus* strains			
UCCSt50	Wild-type strain sensitive to phage P738/host strain for P738		(23)
UCCSt50::pNZ44	Wild-type strain harboring the control/cloning vector pNZ44		This study
F1B	Phage insensitive derivative of UCCSt50/BIM of UCCSt50 generated against phage P738		This study
F2A	Phage-insensitive derivative of UCCSt50/BIM of UCCSt50 generated against phage P738		This study
F1B::pNZ44	F1B harboring the control plasmid, pNZ44		This study
F1B_pNZ44-07020_UCCSt50_	F1B harboring the complementing plasmid		This study
F1B_pNZ44-07020C_UCCSt50_	F1B derivative cured of the complementing plasmid		This study
F2A::pNZ44	F2A harboring the control plasmid, pNZ44		This study
F2A::pNZ44-07020_UCCSt50_	F2A harboring the complementing plasmid		This study
F2A::pNZ44-galE_UCCSt50_	F2A harboring the *galE* complementing plasmid		This study
Brie28	Non-host control strain used in binding studies		(24)
Phage			
P738 (accession number MK911750)	Lytic P738 group phage infecting UCCSt50 used for BIM generation and subsequent assays		(11)
SW13 (accession number MH892362)	*Brussowvirus* of *S. thermophilus* UCCSt50		(31)
Plasmids			
pNZ44	High copy expression vector		(30)
pNZ44-07020	pNZ44 harboring the native *orf*07020 from UCCSt50		This study
pNZ44-galE	pNZ44 harboring the native *orf*06475 from UCCSt50		This study
pHTP9	Green fluorescent protein (GFP) fusion vector used to produce the recombinant GFP-RBP-P738 protein		NZYTech, Portugal

Primers			
Primer	Sequence (5′–3′)	Amplicon size (bp)	Source
RGP			
MSControl_F	gctggtcgtaattacctcg	2,724	(28)
MSControl_R	caacatcttccaaggtacg		
Var1_F	gtatataatgcacaagaggg	895	
Var1_R	gaaactaatcttaagcgttcc		
Fg1_F	gtcatgtgctctaccaattg	1,481	
Fg1_R	gatggagcttataacgttc		
CRISPR loci			
CRISPR1_F	tgctgagacaacctagtctctc	Template dependent	(16)
CRISPR1_R	taaacagagcctccctatcc		
CRISPR2_F	ttagcccctaccatagtgctg	Template dependent	
CRISPR2_R	tagtctaacactttctggaagc		
CRISPR3_F	ctgagattaatagtgcgattacg	Template dependent	
CRISPR3_R	gctggatattcgtataacatgtc		
Plasmid constructs			
pNZ44_F	ctaatgtcactaacctgccccg	Template dependent	(20)
pNZ44_R	gctttatcaactgctgct		
07020_F	aaaaaaccatggaaggaggcactcacatgtctaatccaaccgtttcag	969	This study
07020_R	aaaaaactgcagtacttctcctttcctttacagtaa		
galE_F	aaaaaaccatggaaggaggcactcacatggtggaccgtttggttgaa	972	This study
galE_R	aaaaaactgcagttaatctctatcgtcataacctt		
RBP			
P738-RBPopt_F	tcagcaagggctgagggcggtgaccttctggagcaacagcgat	2001	This study
P738-RBPopt_R	tcagcggaagctgaggttaaatcatgatggtaatctggccacggt		
pHTP9_F	gaatgaaaaacgcgaccacatggtg	RBP amplicon size + 294 bp	NZYTech, Portugal
pHTP9_R	ggttatgctagttattgctcagcg		

**TABLE 3:** Relative efficiencies of plaquing and adsorption levels of phage P738 on its host UCCSt50 and derivatives

Strain	EOP of P738	Adsorption level of phage P738 (%)
UCCSt50	1	83.33 ± 0.37
UCCSt50::pNZ44	0.77 ± 0.12	80.39 ± 0.17
F1B	≤3.6 × 10^−8^	9.08 ± 0.11
F1B::pNZ44	≤3.6 × 10^−8^	14.71 ± 0.26
F1B::pNZ44-07020_UCCSt50_	0.35 ± 0.33	79.41 ± 0.65
F1B::pNZ44-07020C_UCCSt50_	≤3.6 × 10^−8^	14.71 ± 0.07
F2A	≤3.6 × 10^−8^	5.88 ± 0.14
F2A::pNZ44	≤3.6 × 10^−8^	
F2A::pNZ44-07020_UCCSt50_	0.9 ± 0.1	
F2A::pNZ44-galE_UCCSt50_	≤3.6 × 10^−8^	

To evaluate the impact of the *orf*07020_UCCSt50_ mutations on P738 phage infection, adsorption assays of P738 with UCCSt50, BIMs F1B and F2A, and the complemented F1B derivative were undertaken. Indeed, P738 adsorption was reduced by more than ~90% in F1B and F2A compared to the wild-type strain. Furthermore, phage adsorption was restored in the presence of the complementing plasmid for F1B (Table 3), while the plasmid-cured derivative was adsorption deficient. Therefore, it was inferred that the assumed glycosyltransferase activity encoded by *orf*07020_UCCSt50_ is essential to produce the (part of) RGP saccharidic receptor for P738 phage adsorption.

### *orf*07020 is predicted to encode a glycosyltransferase family 2 protein

BLASTP, Pfam, and HHpred analysis of the protein product of *orf*07020_UCCSt50_ revealed significant similarity to family 2 glycosyltransferases involved in cell wall biosynthesis with specific roles in transferring sugar moieties to acceptor molecules. The HHpred analysis returned a 99.94% probability match to GalT1, a glycosyltransferase of *Streptococcus parasanguinis* (32), while BLASTP analysis revealed several significant alignments (E-value 0.0) to *S. thermophilus* glycosyltransferase family 2 proteins. Additionally, Pfam analysis identified a glycosyltransferase domain (PF00535). Interestingly, the mutation in this gene in F2A leads to an amino acid substitution in Asp93 (to an alanine residue), which is one of five conserved residues that are predicted to form the active site of the encoded protein (cd00761; residues 11, 13, 39, 91, and 93 are conserved in GT2 proteins).

**Fig 1:**
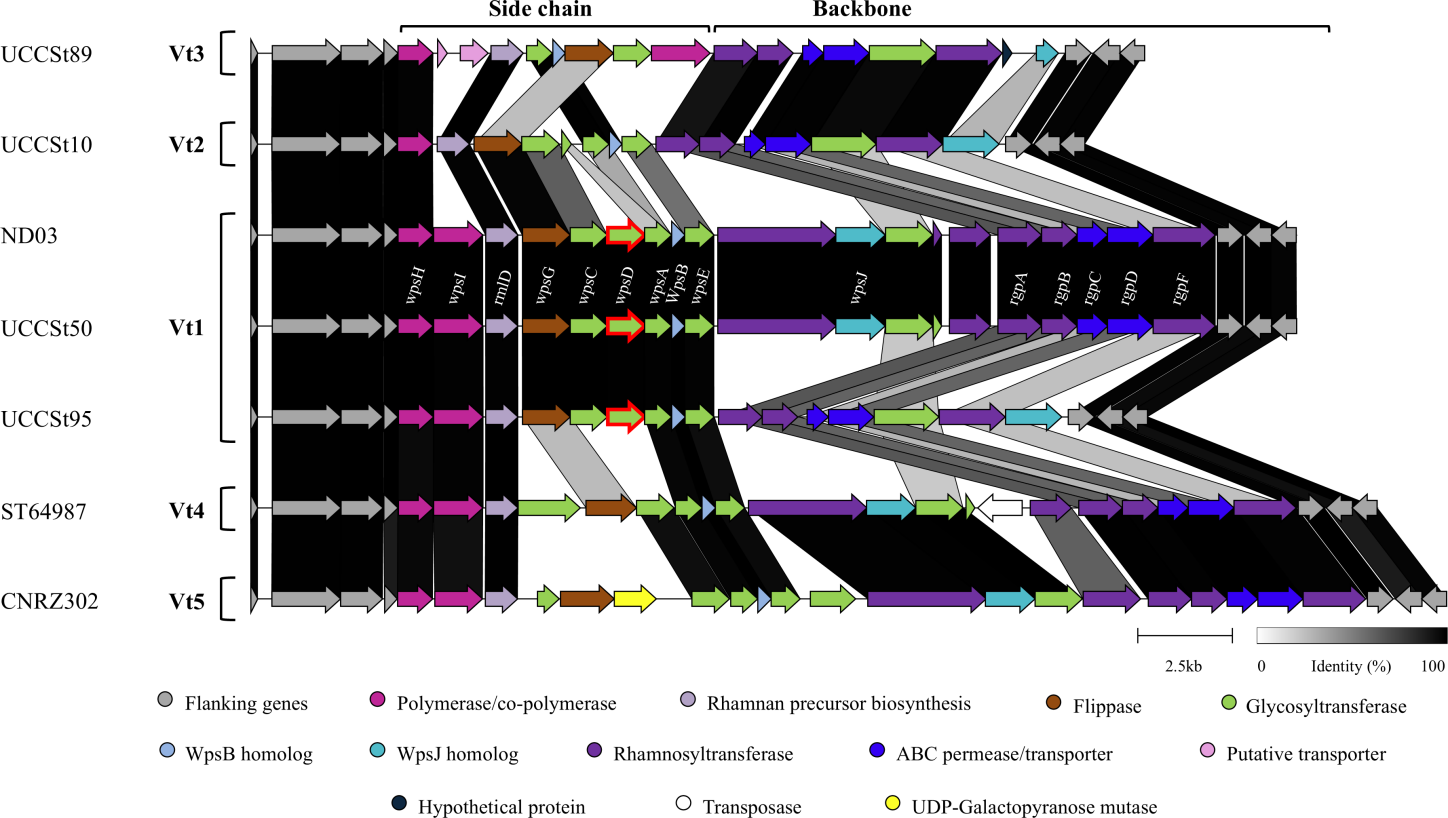
Comparative analysis of *rgp* gene clusters from reference strains. The predicted functions of each of the protein-encoding open reading frames are color coded and indicated at the base of the figure. The *rgp* locus can be split into two distinct regions: the variable side chain (5′) and the more conserved backbone (28) indicated on the figure. The mutated gene (shared between Vt1-type strains) is outlined in red. The mutated gene in BIM B1 (generated in a previous study [23]) is outlined in blue directly downstream of *wpsD*. Regions of homology are joined by blocks of different shades of gray to black and generated using CAGECAT (https://cagecat.bioinformatics.nl/tools/clinker). Gene names (written above the UCCSt50 cluster) were proposed based on comparisons to *Lactococcus lactis* CWPS clusters (35). GenBank accession numbers for strains—UCCSt89 (JANFMW000000000); UCCSt10 (CP065483); ND03 (CP002340); UCCSt50 (CP065477); ST64987 (CP049053); and CNRZ302 (CP065489).

BLASTN analysis of *orf*07020_UCCSt50_ revealed its widespread presence in the genomes of *S. thermophilus* strains with high levels of nucleotide sequence identity (>99% sequence identity across 100% of the query sequence), while it is also found in the genomes of other *Streptococcus* species, including *Streptococcus salivarius, S. parasanguinis,* and *Streptococcus lactarius,* albeit with sequence identity below 95% across the full gene sequence and with lower representation in these species. Specifically, this gene was present in 98 of 517 *S*. *salivarius* genomes available in the NCBI database (at all assembly levels, i.e., contig, scaffold, chromosome, and complete), 50 of 258 *S*. *parasanguinis* genomes (at the same assembly level), and interestingly, it was also present in the only published *S. lactarius* genome. Additionally, in each of these species, the gene was located within the *rgp* locus, identified by the presence of several glycosyltransferase-encoding genes and containing the *rmlD* gene typically found in the gene cluster associated with RGP biosynthesis in streptococci (33). This conservation among species that share overlapping ecological niches, such as the oral cavity (*S. parasanguinis*), food-associated environments (*S. salivarius*), dairy products (*S. thermophilus*), and human milk (*S. lactarius* [34]), suggests a strong evolutionary pressure to maintain the functional role of this gene in RGP biosynthesis, which may contribute to niche adaptation, interspecies interactions, and susceptibility to phages. In contrast, this gene was absent in human-associated or niche-divergent streptococci, including *S. pyogenes*, *S. mutans,* and *S. pneumoniae,* inferring this gene is not required for virulence or infection mechanisms typical of pathogenic streptococci. This gene is unique to S. *thermophilus* strains with a Vt1 *rgp* genotype (Fig. 1). As proposed previously (28), the biosynthesis of the variable side chain structure in UCCSt50 is initiated by the priming glycosyltransferase and its associated activator. *Orf*07020_UCCSt50_ (previously termed *scbD*_UCCSt50_) is believed to be involved in the subsequent elongation (addition of monosaccharides) of the side chain structure. However, the exact nature of the substrate and order of assembly are unknown (28). Based on these observations, we speculate that mutations in this gene impact the sequential addition of monosaccharide residues to the lipid-linked GlcNAc intermediate during UCCSt50 RGP side chain biosynthesis, potentially altering the structure or assembly of the final side chain structure, thereby affecting phage sensitivity.

It has previously been demonstrated that P738, as well as the other identified member of the P738 genus, D4446, possesses broad host ranges (11). We speculate that the P738-sensitive strains applied in this study possess a Vt1 genotype. A high prevalence of Vt1-type strains has also been reported, with Vt1-type *S. thermophilus* strains in an industrial strain collection being reported at a level of 50% (RGPA and RGPB possess a Vt1 genotype) (22). Similarly, in a recent study, approximately 17% of all isolates from a range of fermented dairy products possessed a Vt1 genotype (24), while 85% of *S. thermophilus* isolates from Siciliano PDO cheeses were shown to display the Vt1 genotype (36). These studies highlight the prevalence of strains possessing this genotype in both industrial and artisanal contexts. Furthermore, there are currently 156 *S*. *thermophilus* genome sequences available in the NCBI database (at the chromosome or complete genome assembly level); BLASTN analysis of the unique Vt1 gene against these genomes returned 61 sequence hits (all with E values of 0.0), representing 39.1% of the total publicly available genomes. These results reinforce the prevalence of Vt1-type strains in *S. thermophilus* populations and underscore the industrial importance of our findings, as they reveal a P738 phage infection profile dependent on the unique Vt1 gene, highlighting a potential vulnerability in industrial strains.

### Preparation and structural analysis of the RGP from the P738 phage-resistant derivative of UCCSt50, F1B

To verify if mutations within *orf*07020_UCCSt50_ affect the RGP structure (as was previously observed for the priming glycosyltransferase-encoding gene [23]), we prepared and analyzed the RGPs of the parent strain, UCCSt50 (23), and the BIM F1B. Compositional analysis revealed the presence of rhamnose (Rha), glucose (Glc), N-acetylglucosamine (GlcNAc), and galactose (Gal) for both the wild-type and the phage-resistant derivative. However, the amount of Rha in the RGP of the mutant was lower compared to the wild type (with the ratio ~3:4 BIM:WT).

**Fig 2:**
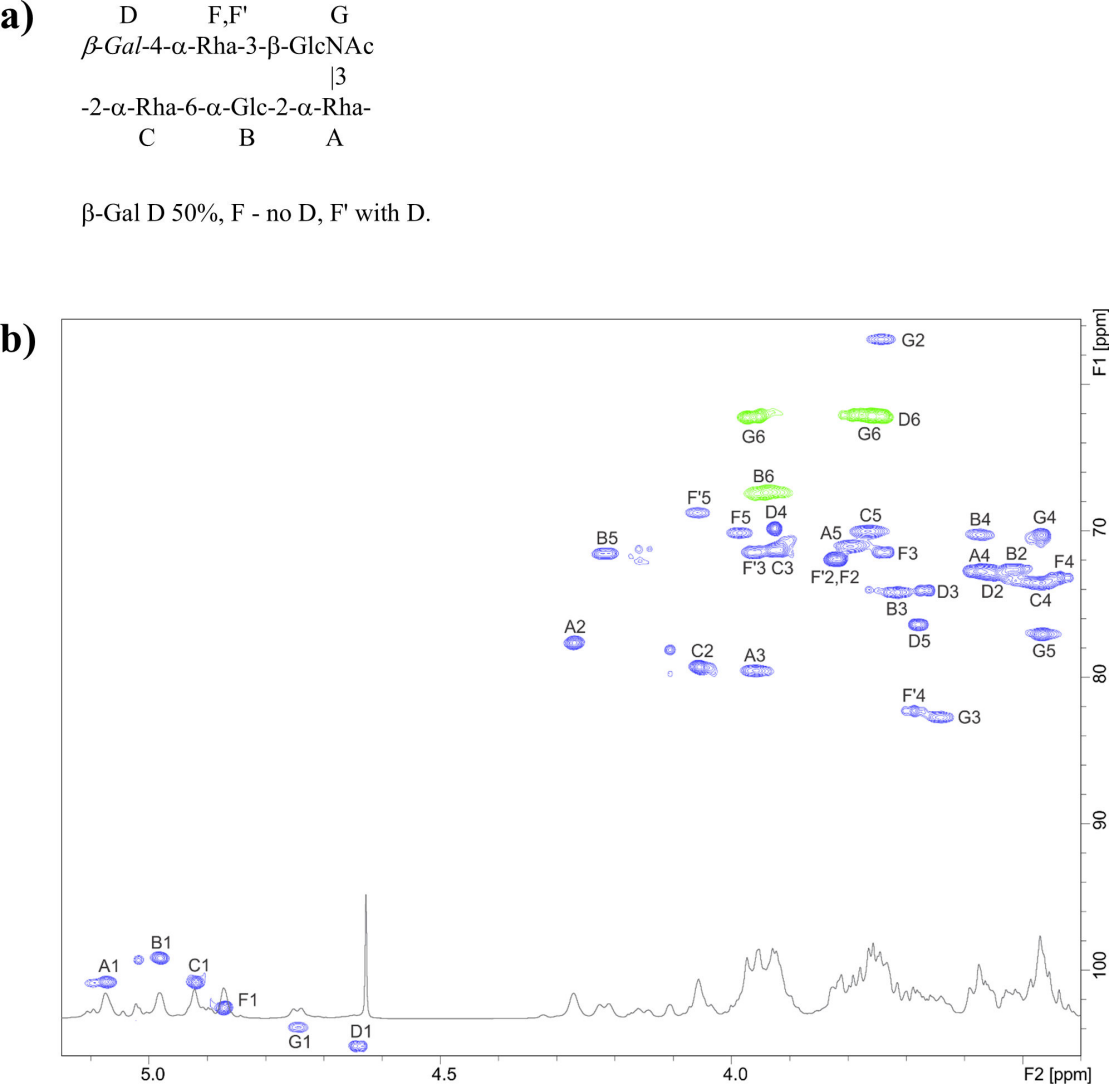
Structure of the repeating unit (**a**) and fragments (**b**) of ^1^H-^13^C HSQC and ^1^H NMR spectra of the RGP from the BIM F1B. In the HSQC spectra (**b**), different colors (green and blue) distinguish between positive and negative NMR signals.

**Fig 3:**
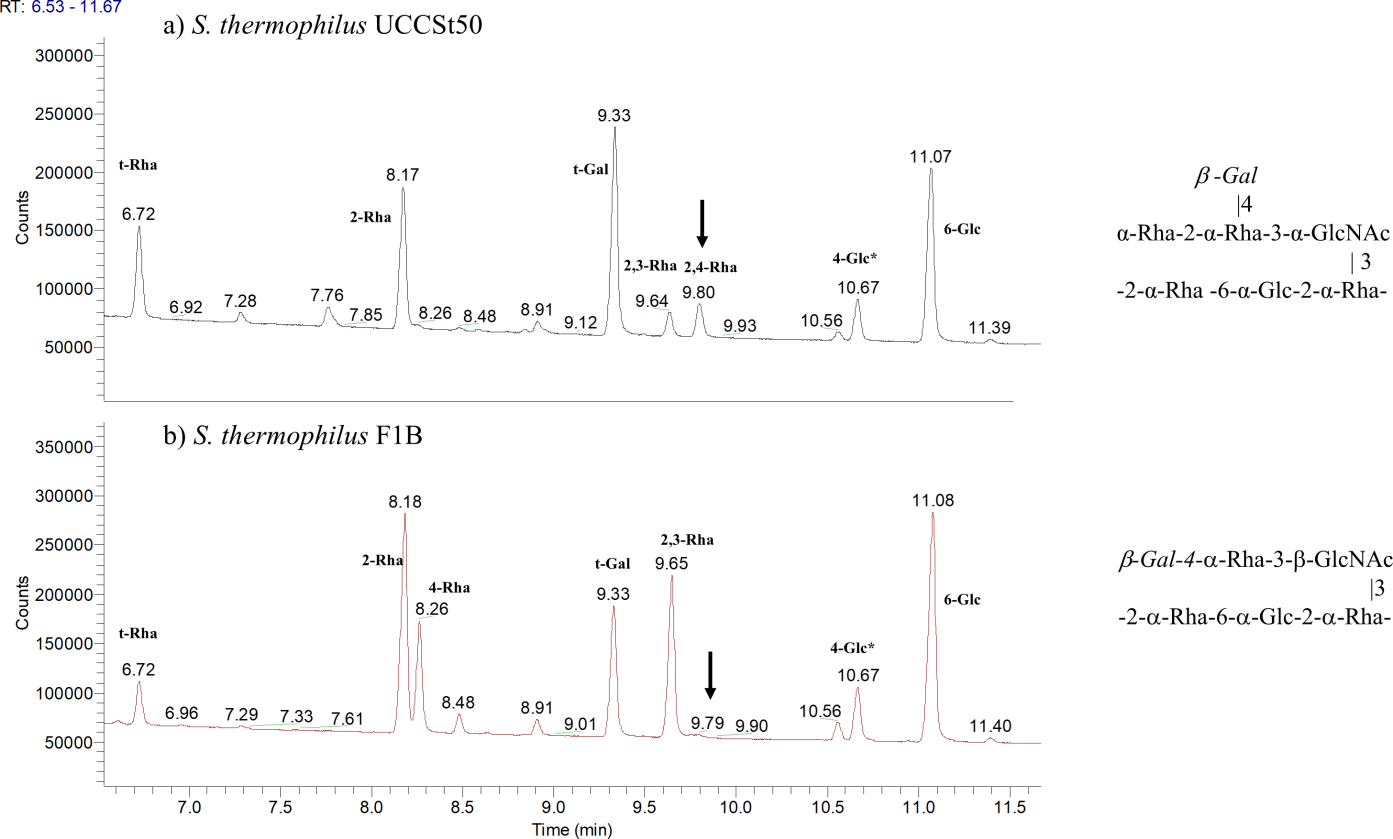
Methylation analysis profiles of the RGP preparations of the wild-type *S. thermophilus* UCCSt50 (**a**) and its P738-resistant derivative F1B (**b**) and the associated RGP structures. In the absence of the terminal Rha residue, the branched 2,4-Rha becomes 4-Rha (corresponding peaks are marked with arrows). Approximately 50% of the terminal β-Gal residue is absent in F1B RGP. Peaks labeled with an asterisk represent polysaccharide(s) other than RGPs.

Detailed 2D NMR analysis of the RGP of the BIM F1B (Fig. 2; Table S4) revealed that the backbone structure is composed of trisaccharide-repeating units (–2-α-Rha-6-α-Glc-2-α-Rha–), with linear tri- or disaccharide side chains on the α-Rha residue A (Fig. 2). Compared to the RGP of the wild-type strain UCCSt50, the terminal α-Rha residue is absent in all RGP side chains, and the β-Gal residues are absent in 50% of the RGP side chains (Fig. 3). The results of methylation analysis agreed with the proposed structure (Fig. 3). We propose that the absence of the terminal Rha (-α-Rha-2 moiety in WT structure, Fig. 3) is associated with a reduced efficiency of incorporating the Gal side chain.

Therefore, we conclude that *orf*07020_UCCSt50_ is a glycosyltransferase-encoding gene responsible for the addition of the terminal rhamnose residue to the side chain structure. It may be inferred that this alteration to the RGP structure results in resistance to phage P738.

### The RGP side chain structure is required for P738 phage infection

Alterations to the CWPS structures of *Lactococcus lactis* and *S. thermophilus* have been implicated in phage resistance, primarily by disrupting phage adsorption at the cell surface (20, 23, 37, 38). Recent work has demonstrated that the RGP side chain structure of *S. thermophilus* UCCSt50 is also essential for host adsorption by the *Brussowvirus* SW13 (23). To establish if the impact of the *rgp* locus mutations on phage adsorption was similar or distinct in these two members of distinct phage genera, the sensitivity of UCCSt50 and selected BIMs to SW13 (*Brussowvirus*) (31) and P738 was assessed (Table 4; all reported results are the average of at least triplicate biological assays). The wild-type UCCSt50 strain was, as expected, confirmed to be susceptible to either of these two phages, while BIM B1 (generated in a previous study and which lacks the entire RGP side chain structure [23]) was resistant to both phages, confirming the necessity of the RGP tetrasaccharidic side chain for infection. Notably, F1B, which has a structurally altered side chain, and F2A are susceptible to SW13 (EOP = 0.46 ± 0.08 and 0.69 ± 0.16, respectively) and resistant to P738. These results highlight the distinct structural requirements for SW13 and P738 (partial vs complete side chain structure), i.e., while SW13 can infect strains with a tri- or disaccharidic side chain structure, P738 infection is dependent on the presence of the intact RGP structure (comprising a backbone composed of trisaccharide-repeating units (–2-α-Rha-6-α-Glc-2-α-Rha–) and a tetrasaccharide side chain containing GlcNAc, Rha, Glc, and Gal residues (23). This underscores the role of specific host-encoded glycan features in mediating phage–host interactions and establishes that complete structural integrity or a specific part of the RGP side chain is required for P738 infection.

**TABLE 4:** Relative efficiencies of plaquing of phages SW13 (*Brussowvirus*) and P738 on their primary host UCCSt50 and derived BIMs

Strain	EOP of SW13	EOP of P738
UCCSt50	1	1
B1	≤1.6 × 10^−6^	≤1.17 × 10^−7^
F1B	0.46 ± 0.08	≤1.17 × 10^−7^
F2A	0.69 ± 0.16	≤1.17 × 10^−7^

### RGP mutations cause growth aberrations

**Fig 4:**
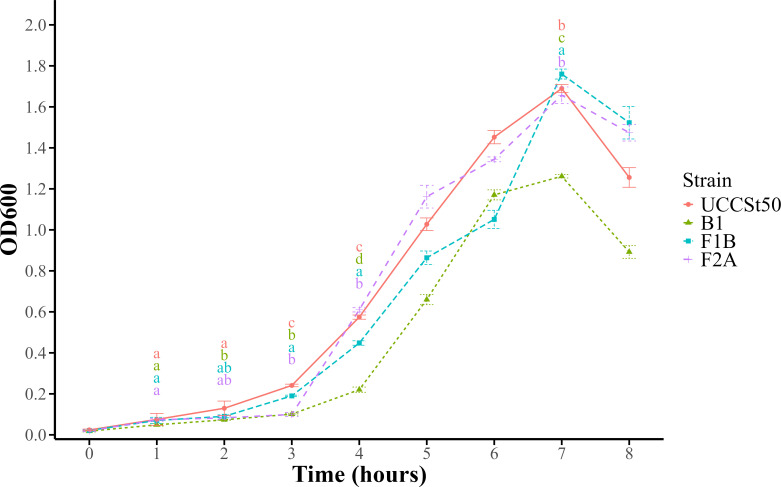
Growth in liquid LM17 medium of *S. thermophilus* UCCSt50 and its phage-resistant derivatives. Each point represents the mean value at an optical density of 600 nm (OD_600_) of three replicates. The standard deviations for each value are represented by error bars. One-way ANOVA followed by Tukey’s honestly significant difference test was performed at hours 1, 2, 3, 4, and 7 using the multcompLetters function in R to assign group letters based on the adjusted *P*-values from Tukey’s test. Compact letter displays indicate significant differences between strains (*P* < 0.05); letters are colored by strain; strains sharing a letter are not significantly different.

It has previously been shown that alterations to cell wall polysaccharide structures in LAB can have an impact on bacterial growth kinetics, including delayed growth and reduced overall cell density (37, 39, 40). Similar findings have been observed in pathogenic streptococcal species. For example, deficiency of RgpG affects cell division and biofilm formation in *Streptococcus mutans* (41), while mutants of *rgpE* elicit a reduced growth rate compared to the wild-type strain in the same species (42). Interestingly, modifications to the RGP structure in *Streptococcus pyogenes* and *Enterococcus faecalis* (the RGP structures are termed GAC and EPA, respectively) were not observed to affect bacterial growth yet significantly impact virulence (43–45). To determine the impact of RGP-associated mutations on planktonic growth and possible sedimentation in this study, growth characteristics of UCCSt50 and its phage-resistant derivatives over an 8-hour period were monitored using OD readings at a fixed point on the culture-containing tube (Fig. 4). All tested BIMs exhibited a slower growth rate during the early phases compared to the wild-type strain. Notably, B1 demonstrated the longest lag phase (defined in this study as the period between hours 1 and 4), growing consistently slower than the other tested BIMs and failing to reach the peak OD observed in the wild type (or BIMs lacking the terminal rhamnose) (Fig. 4). Strains F1B and F2A showed an initial lag in growth but ultimately reached optical densities comparable to that observed for UCCSt50. All strains, including the wild type, displayed a rise-and-fall growth profile (Fig. 4), which is expected given the visual observation of the sedimenting phenotype commonly associated with *S. thermophilus* BIMs that possess altered CWPS structures (20, 23).

### Functional assignment of the receptor binding protein of phage P738

In order to further assess the molecular interactions involved in host recognition by P738, we wanted to validate the functionality of the proposed RBP of this phage. As a member of the most recently recognized genus of *S. thermophilus* phages, P738 has not been extensively studied in terms of host recognition or protein functions. However, several open reading frames (ORFs) have been identified with predicted roles in phage morphogenesis and host interaction (11). Orf16_P738_ has been annotated as encoding the tail tape measure protein, Orf17_P738_ as the distal tail protein (Dit), and Orf18_P738_ as the Tal protein. HHpred analysis of a previously predicted structural protein (protein id = QDP43720.1) encoded by a gene located downstream of the Tal-encoding gene (11) revealed a phage RBP-like domain, identifying it as the putative RBP of phage P738. HHPred analysis revealed the presence of several high probability hits to receptor binding proteins or tail fiber proteins of phages, including T4 (99.7% PDB_5HX 2_I gp10 baseplate wedge protein), TP901-1 (96.0% probability PDB_4IOS_B receptor binding protein), and *Staphylococcus* phage phi812 baseplate protein (98.6% probability PDB_9EUF_q). These high-probability hits are all located within the C-terminal region, which is predicted to represent the so-called “head” or host binding domain of the receptor binding protein. Alignment of the protein sequences of the RBP sequences of TP901-1 and P738 also highlighted that the most significant region of sequence conservation was located within the C-terminal region of the respective proteins, with 22 conserved amino acids in this region likely accounting, in part, for the structural conservation of the RBP head domains of these proteins.

**Fig 5:**
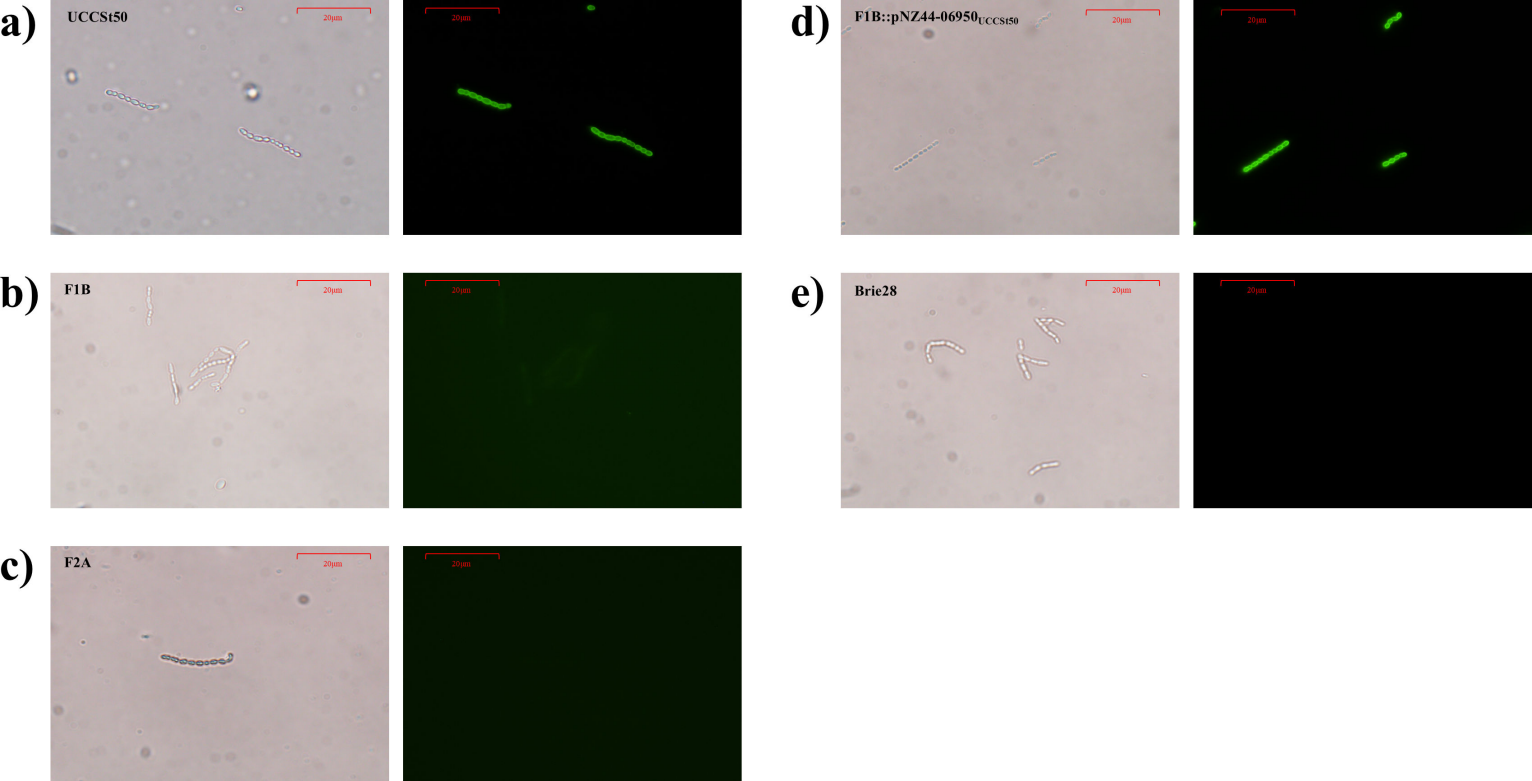
Brightfield (left panels) and fluorescent labeling images (right panels) of the (**a**) parent strain UCCSt50, its phage-resistant derivatives (**b**) F1B and (**c**) F2A, (**d**) the genetically complemented strain F1B::pNZ44-07020_UCCSt50_, and (**e**) a non-host strain (Brie28) using 30 µg protein at 100× magnification. The scale bars are indicated in the individual images.

To establish if the proposed RBP is indeed responsible for host recognition, a green fluorescent protein (GFP)-tagged derivative of the predicted RBP was constructed and applied in host binding studies using the wild-type strain, its P738-resistant derivatives, and a non-host strain (Fig. 5). Binding assays using 30 µg of the heterologously expressed GFP-RBP_P738_ fusion protein exhibited complete cell surface binding of the cognate host UCCSt50, consistent with the predicted functional activity of the P738 RBP in host recognition. In contrast, a significantly reduced fluorescence signal (and thus binding) was observed for the BIMs, indicating reduced host binding consistent with the adsorption assay findings. RBP binding was restored in the complementing strain (F1B::pNZ44-07020_UCCSt50_), with observed fluorescence comparable to the parent strain UCCSt50. Binding was not observed (no fluorescence) for the non-host control strain, *S. thermophilus* Brie28, which possesses a distinct *rgp* genotype, i.e., V2B2 (24). These results demonstrate that the host-encoded RGP structure in UCCSt50 acts as the specific receptor for P738 phage and confirms the role of this gene product as the primary RBP of phage P738.

## DISCUSSION

Deciphering the host-encoded receptors of dairy phages has long been recognized as a cornerstone of generating starter cultures to include strains that are resistant to phages, thereby increasing the resilience of dairy fermentations (46–48). While previous research has highlighted the role of EPS in mediating phage adsorption for members of the *Piorkowskivirus*, *Moineauvirus,* and *Vansinderenvirus* genera (12, 20–22, 24), the present study provides evidence supporting the involvement of the RGP as the host-encoded receptor for P738 phages in addition to the *Brussowvirus* SW13 (23). Our findings highlight that host recognition and binding by P738 is underpinned by *orf*07020_UCCSt50_, which is located within the *rgp* locus and encodes a glycosyltransferase. This gene is unique to dairy streptococcal strains possessing a Vt1 genotype and the associated RGP structure that is now confirmed as the receptor for both P738 and the *Brussowvirus* SW13. In the present study, two BIMs (among a panel of 20 BIMs) were selected for detailed characterization; however, it is likely that genome sequencing of additional BIM genomes may reveal additional insights into the genes underpinning host binding processes and represents an area of future research.

It has previously been demonstrated that the two currently known P738 phages (P738 and D4446) have broad host ranges, which is not typical among dairy phages (11). In this study, a BLASTN search against 156 publicly available *S. thermophilus* genomes revealed the presence of the unique Vt1 gene in 61 strains, highlighting the possible prevalence of Vt1-type strains within the species. While it is not possible to infer that all *S. thermophilus* strains exhibiting a Vt1 genotype will be bound and subsequently infected by P738-type phages without experimental validation, the widespread presence of Vt1 genotype strains represents a risk factor for such scenarios. Binding studies exploring the full binding potential of P738 phage receptor binding proteins are currently under investigation (and which are beyond the scope of this manuscript) and will serve to address this knowledge gap. CWPS biosynthesis elicited through the functions of genes within the *cwps* locus of *L. lactis* is well characterized. The lactococcal CWPS is organized into distinct functional regions, including a conserved region responsible for the synthesis of a rhamnan backbone component and a highly variable region encoding the surface-exposed oligosaccharide or polysaccharide pellicle (PSP) (46, 49). A comprehensive CWPS biosynthesis scheme has been proposed where the rhamnan and PSP side chains are synthesized independently from separate lipid-sugar precursors, then joined extracellularly by a GT-C fold glycosyltransferase (35). The functional assignment of genes was based on structural similarity prediction by HHpred analysis combined with transmembrane helix prediction (35). In contrast, the *rgp* cluster of *S. thermophilus* remains less well defined. Although a proposed model for *rgp* biosynthesis has recently been developed (28), functional annotation of genes within the cluster is still emerging. In this study, we assigned gene function guided by approaches used for CWPS classification in *L. lactis*. This includes the analysis of predicted transmembrane domains to infer roles in membrane-associated steps of the pathway, and the identification of glycosyltransferases based on conserved GT2 and GT4 family domains. The resulting framework aligns with the dual-pathway model observed in *L. lactis* and adds to the proposed pathway previously developed for *S. thermophilus rgp* loci.

Genetic complementation of the two phage-resistant derivatives with the native *orf*07020_UCCSt50_ restored P738 phage sensitivity, suggesting a direct link between this gene and phage susceptibility. This is consistent with previous studies in *S. thermophilus* and other lactic acid bacterial species (*L. lactis*), where glycosyltransferases involved in CWPS biosynthesis have been implicated in host recognition and adsorption (21–23, 38, 49). Adsorption assay results demonstrate that mutations in *orf*07020_UCCSt50_ significantly reduced phage adsorption by P738, a phenotype corroborated by fluorescence-based RBP labeling assays. Similar observations have been made in *S. mutans*, for which the sero-specific region of the RGP cluster plays a crucial role in phage adsorption (50), and in *E. faecalis*, where the enterococcal polysaccharide antigen cluster determines phage susceptibility (51).

Compositional analysis of the RGP in the wild-type and phage-resistant derivative F1B revealed the complete loss of the terminal rhamnose saccharide, as well as the absence of approximately 50% of the β-Gal residues in the RGP side chain structure, which is surface-exposed. Previous studies have demonstrated that mutations in the predicted priming glycosyltransferase of the *rgp* gene cluster are associated with an absence of the side chain structure and complete resistance to phage SW13 (*Brussowvirus*) (23). In the current study, we confirm that this mutant (B1) is also resistant to P738, which is consistent with our finding that P738 requires (part of) the RGP side chain for adsorption. Interestingly, we demonstrated in the current study that SW13 can infect BIMs that lack the terminal rhamnose monosaccharide of the RGP side chain with almost equal efficiency to the parent strain, while P738 cannot. This result provides the first insight into the minimal RGP structural requirements for phage infection of the P738 and *Brussowvirus* genera and suggests that P738 has a strict requirement for the complete or distinct/specific part of the tetrasaccharide side chain for binding, while SW13 requires at most three of the four component monosaccharides for successful adsorption. While the saccharidic receptor moiety for representatives of each of the five dairy streptococcal phage genera is now defined, future studies may focus on deriving the exact and minimal receptor that these phages bind to, which in turn will improve the predictions of phage sensitivity of strains that are applied in dairy fermentations. These findings could play a crucial role in developing phage-robust cultures and strategies to enhance the stability of industrial dairy fermentations.

It has recently been shown that the reduction in the production of cell wall polysaccharides may allow bacterial starter cultures to evade phage infection (52). Here, amino acid substitutions in a cell wall-associated glycosyltransferase may contribute to the fine-tuning and reduction in the production of RGP side chain with an associated (and possibly reversible) cessation in phage recognition. Although attempts were made to isolate phage escape mutants of P738 capable of infecting the BIMs, none were recovered in this study. Nevertheless, it is likely that repeated exposure of BIMs with non-synonymous missense mutations to phages at an industrial scale would select for such phage mutants. Alternatively, where phage pressure is removed or significantly reduced, the BIMs may revert to the wild type as observed in *Lactococcus cremoris* (52). One of the most significant challenges with dairy streptococcal phages is the inability to reproducibly achieve high titer phage lysates as is possible within other bacterial host species. The inability to achieve high titers (beyond 10^7^–10^8^ pfu/mL) hampered our efforts to challenge the BIMs with a sufficiently high number of phages to generate phage escape mutants. It is also possible that several mutations are required for P738 to overcome the modified cell surface, and this may not be possible in a single round of exposure to the phage. Therefore, future studies may focus on repeated longitudinal studies to achieve such mutations notwithstanding the issue of the low titer of these phages. Future studies pertaining to the isolation of such phage escape mutants will facilitate a deeper understanding of the amino acids within the receptor-binding or other adhesion proteins that are involved in the saccharide-binding process.

Here, we show that RGP-associated mutations in *S. thermophilus* UCCSt50 negatively affect growth, with mutant B1 showing the longest lag phase and mutants F1B and F2A exhibiting milder growth defects. These findings suggest that the extent of structural disruption to the RGP correlates with the severity of the growth defect. The pronounced growth impairment observed in B1, which completely lacks the side chain, contrasts with the milder growth impact observed for F1B, which has a structurally altered RGP side chain and F2A. This supports the notion that even subtle modifications to the CWPS structure can impact bacterial fitness as well as phage sensitivity.

Recently, we have shown that P738 is affected by several anti-phage systems encoded on *S. thermophilus* genomes, including GAO19, Hachiman, SoFic, AbiD, and AbiE (53). Therefore, while the presence of a suitable receptor is critical to the initial interactions of P738 phages with their host, it is imperative that the internal anti-phage landscape is also compatible with phage replication. Since P738 phages have only been identified recently, it is unclear how much exposure these phages may have had to the anti-phage repertoire of *S. thermophilus* or if their genomes encode counter defenses to CRISPR-Cas, restriction/modification, and the plethora of other antiphage systems. Anti-CRISPR and methyltransferase-encoding genes have been identified on the genomes of other genera of dairy streptococcal phages (9, 54, 55). Therefore, future studies may focus on the identification of such counter-defense systems in P738 phages to enhance current understanding of the co-evolution of these phages with their hosts.

In conclusion, the current study has identified the specific RGP component required by P738 for host recognition and binding, while the predicted receptor binding protein of phage P738 has been functionally proven. These insights enhance our understanding of the mechanisms by which dairy streptococcal phages interact with their hosts and the specificity of streptococcal phages. This information will be pivotal in informing the development of phage-resistant bacterial strains and the selection of strains to enhance the robustness of starter cultures, thereby improving the sustainability of dairy fermentation practices. Further investigations into the biochemical structures of additional *S. thermophilus* strains and the role of additional genes within the *rgp* cluster may facilitate more detailed correlations between the *rgp* genotype and the associated RGP chemical composition and structures and predictable outcomes in dairy fermentations.

## MATERIALS AND METHODS

### Bacterial strains and bacteriophages

Bacterial strains and bacteriophages used in this study are listed in Table 2. *S. thermophilus* strains were routinely grown overnight at 42°C from single colonies or bacterial stocks stored at −20°C (25% [wt/vol] glycerol) in M17 (Sigma, USA) supplemented with 0.5% lactose (Sigma, USA). For *S. thermophilus* strains containing plasmids (UCCSt50::pNZ44, F1B::pNZ44, F1B::pNZ44-07020_UCCSt50_, and F2A::pNZ44-galE_UCCSt50_), chloramphenicol (Sigma, USA) was added to LM17 at a final concentration of 3 µg/mL in agar and 5 µg/mL in broth.

### Bacteriophage assays

Phage P738 was propagated in fresh LM17 broth supplemented with 10 mM CaCl_2_. The LM17 broth was inoculated with 1% of a fresh overnight culture of *S. thermophilus* UCCSt50, and 10 µL P738 phage lysate was added and incubated until visible lysis was observed (approximately 6 hours) or overnight at 42°C. Phage lysates were filtered (0.45 µm, Sarstedt, Germany) and stored at 4°C.

Bacteriophages were enumerated using the standard overlay method (56), in which LM17 medium was supplemented with 10 mM CaCl_2_ and 10 g/L (solid agar) or 4 g/L (semi-solid agar) technical agar (Neogen, USA). Briefly, 400 µL of a fresh UCCSt50 culture grown overnight and 10 µL of the P738 phage lysate (≥10^7^ PFU/mL) were mixed in 4 mL semi-solid LM17 agar and poured onto LM17 solid agar before incubation at 42°C overnight.

### Bacteriophage-insensitive mutant generation

BIMs of *S. thermophilus* UCCSt50 were generated using a standard plaque assay protocol (56). Briefly, 400 µL of a fresh UCCSt50 overnight culture and 10 µL of phage P738 lysate (at a titer of ≥10^7^ PFU/mL) were mixed in 4 mL semi-solid LM17 agar (4 g/L agar) supplemented with 10 mM CaCl_2_ and poured onto LM17 agar plates and incubated anaerobically (Anaerocult A, Millipore, Germany) at 42°C for 24 hours. Surviving colonies on the semi-solid agar were picked and passaged three times in fresh LM17.

All generated BIMs were screened for insensitivity to phage P738 using the spot and plaque assay methods (as described above). BIMs exhibiting complete or incomplete P738 resistance were confirmed as UCCSt50 derivatives by *rgp* PCR (28) (Table 2) and by visually comparing the amplicons of the CRISPR 1, 2, and 3 repeat spacer loci (16) (Table 2) on a 1% agarose gel.

### DNA extraction, genome sequencing, and SNP analysis

Genomic DNA of strain UCCSt50 and two BIMs (F1B and F2A) was extracted using a modified protocol of the Nucleobond AXG 100 with Buffer set III (Macherey-Nagel, Germany). This choice of BIMs was based on a previous observation by our group that SW13-resistant derivatives of UCCSt50 produced mutations strictly in the priming glycosyltransferase-encoding gene, which exhibited a complete phage-resistance phenotype. Therefore, to maximize the potential to identify distinct mutations, the two BIMs with incomplete phage resistance were selected for further characterization. DNA extraction was conducted using a modified protocol of the Nucleobond AXG100 kit with Buffer Set III (Macherey-Nagel, Germany). Initially, 40 mL cells grown to an OD_600_ of ~1.0 were pelleted by centrifugation (5,000 × *g* for 20 minutes), followed by pre-treatment of the cell pellets with lysozyme (at a final concentration of 0.8 mg/mL; Sigma, USA) and mutanolysin (at a final concentration of 50 units/mL; Sigma, USA) at 37°C for 1 hour. Proteinase K (supplied with Buffer set III) was subsequently added (at a final concentration of 100 µg/mL), and samples were incubated at 50°C for an additional hour. The extraction protocol was completed according to the manufacturer’s protocol using AXG 100 columns. DNA was resuspended in 10 mM Tris buffer (Fischer Scientific, USA) prior to whole-genome sequencing.

Bacterial genome sequencing was performed by Plasmidsaurus (USA) using Oxford Nanopore Technology (UK) long-read paired with Illumina short-read sequencing. Long-read sequencing was conducted on a PromethION P24 platform with R10.4.1 flow cells, and libraries were prepared using the Rapid Barcoding Kit 96 V14. Basecalling was performed in “super-accurate mode” using the ont-doradod-for-promethion algorithm version 7.4.12, with ONT reads meeting a minimum *Q*-score of 10 and adapters trimmed via MinKnow (57). Illumina (USA) short-read sequencing was performed on an Illumina NextSeq2000 platform using libraries prepared with the SeqWell ExpressPlex 96 kit, using paired-end reads 2 × 150 bp chemistry. Illumina-derived forward and reverse reads were aligned to the ONT assembly using BWA-MEM (58), which clips low-quality sequences from the ends of reads. Polypolish version 6.0 (59) was used to generate the final, polished genome assembly, which was subsequently annotated using Bakta version 1.6.1 (60) with default parameters.

The quality or completeness of genome assemblies was evaluated with the microbial genomes atlas (MiGA) webserver (61). To verify that no modifications had been made to the BIM CRISPR (1, 2, and 3) loci spacer content or number, all sequenced genomes were uploaded to CRISPRCasFinder (https://crisprcas.i2bc.paris-saclay.fr/CrisprCasFinder/Index) (62), and CRISPR spacer arrays were analyzed. A single nucleotide polymorphism analysis of BIM F1B and F2A genome sequences against the genome of the parent strain UCCSt50 was performed using the Bowtie2 alignment tool (63) and SAMtools (64).

### Molecular cloning and complementation studies

The native *orf*07020_UCCSt50_ and *orf*06475_UCCSt50_ were amplified with Phusion Hot Start II High-Fidelity PCR Master Mix (Thermo Fisher Scientific, USA) using primer combinations 07020_F and 07020_R and galE_F and galE_R (Table 2). The resulting amplicons were purified using the GenElute PCR Clean-Up Kit (Sigma, USA). The amplicons and pNZ44 cloning vector were digested with NcoI and PstI fast digest enzymes (Thermo Scientific, USA) at 37°C for 30 minutes. All digests were purified (GenElute PCR Clean-Up Kit; Sigma, USA) before ligation was performed overnight at room temperature using T4 DNA ligase (Promega, UK). The ligation mixture was dialyzed against sterile, distilled water for 20 minutes before being introduced into competent *Escherichia coli* EC101 cells. Briefly, 5 µL of the ligation mixture was added to 45 µL of competent EC101 cells in a pre-chilled electroporation cuvette held on ice. The cells were subjected to a 2.5 kV single pulse before 950 µL pre-warmed LB was added to the cuvette. Recovery was performed at 37°C with agitation (150 rpm) for at least 1 hour before plating on LB agar supplemented with 10 µg/mL chloramphenicol and incubated overnight at 37°C. The resulting presumptive transformants were screened by colony PCR using pNZ44_F and pNZ44_R (Table 2), and “positive” clones were grown overnight at 37°C in LB supplemented with 10 µg/mL chloramphenicol. Plasmid DNA was subsequently extracted from selected transformants using the GeneJet plasmid miniprep kit (Thermo Scientific, USA), and the integrity of the construct was validated by Sanger sequencing (Eurofins, Germany) before being introduced into BIM F1B or F2A.

Fresh competent cells of BIM F1B and F2A were prepared as previously described (23) with the following modifications: cells were grown in LM17 supplemented with 0.3% glycine and a sucrose concentration of 0.25 M in place of 0.5 M. Briefly, 100 µL of cells and 10 µL of plasmid DNA were mixed and left on ice for 30 minutes before a heat shock was applied at 42°C for 45 seconds followed by holding on ice for 2 minutes (65). The mixture was transferred to a pre-chilled electroporation cuvette before applying a single 2.0 kV pulse followed by the immediate addition of 900 µL HJL recovery media (3% tryptone, 1% yeast extract, 0.5% KH_2_PO_4_, 0.2% beef extract, 0.5% lactose, 20 mM CaCl_2_, and 200 mM MgCl_2_) and incubating at 42°C for a minimum of 4 hours. Cells were plated on LM17 agar supplemented with 3 µg/mL chloramphenicol and incubated at 42°C for 24–72 hours. Positive transformants were verified to contain the recombinant plasmids by colony-based PCR using pNZ44_F and pNZ44_R primers (Table 2) and confirmed by Sanger sequencing (Eurofins, Germany).

The complementing plasmid was cured from F1B::pNZ44-07020_UCCSt50_ by novobiocin treatment and serial passaging in the absence of chloramphenicol. Fresh LM17 broth was inoculated with 1% of an overnight culture of the complemented strain F1B::pNZ44-07020_UCCSt50_ and allowed to grow at 42°C for 2.5 hours. Novobiocin (Sigma, USA) was added to the culture at a final concentration of 10 µg/mL followed by an additional 5-hour incubation. The culture was then plated in the presence or absence of chloramphenicol for several passages. The cured derivative, F1B::pNZ44-07020C_UCCSt50_, was tested for P738 phage sensitivity. All assays were performed in at least triplicate, and the average and standard deviation were calculated using the standard Student’s *t*-test in MS Excel.

### Efficiency of plaquing and adsorption assays

The efficiency of plaquing of phage P738 on all tested strains listed in Table 2 was determined using the standard overlay method described above to assess the role of *orf*07020_UCCSt50_ in the P738 phage infection process. The relative EOP of phages on the listed strains was determined by dividing the observed titer of the phage on a given BIM/complement/cured host by that on the parent strain. All assays were performed in triplicate, and averages and standard deviations were calculated in MS Excel using a Student’s *t*-test.

Adsorption assays were performed based on a previously adapted protocol (9). A volume of 10 mL LM17 broth was inoculated with the appropriate *S. thermophilus* strain from a fresh overnight culture and grown at 42°C until an OD_600_ of 0.6–0.7 was achieved. A 500 µL volume of the growing culture was transferred to a microcentrifuge tube and centrifuged at 5,000 × *g* for 10 minutes to harvest the cells. The cell pellet was resuspended in 500 µL of SM buffer (100 mM NaCl, 10 mM Tris-HCl [pH 7.5], 20 mM CaCl_2_, and 10 mM MgSO_4_) before an equal volume of P738 phage lysate (≤10^5^ PFU/mL) was added to the cells or 500 µL of SM buffer (negative control). The mixture was incubated at 42°C for 15 minutes and centrifuged at 15,000 × *g* for 5 minutes to remove bacterial cells before removing the residual phage-containing supernatant for enumeration as described above. The adsorption levels (as a percentage of the total number of phages present) were determined using the following formula: ([control phage titer − free phage titer in supernatant]/control phage titer) × 100. All assays were performed in triplicate, and averages and standard deviations were calculated as described above.

### Comparison of bacterial growth behavior

Bacterial strains were inoculated (2%, vol/vol) into 5 mL of LM17 broth in a clear, sterile round-bottom tube (Corning Science, Mexico). Growth was recorded by placing the tubes in a Jenway 7200 visual spectrophotometer (Bibby Scientific, UK) and measuring at an optical density of 600 nm (OD_600_). Tubes were incubated at 42°C, and OD_600_ readings were taken hourly over an 8-hour period. Growth of each strain was measured in triplicate. OD_600_ readings were plotted as mean ± standard deviation using R (version 4.4.1) (R Core Team, 2024) and the ggplot2 package (66).

To compare growth differences between strains at each time point, one-way ANOVA was performed, followed by Tukey’s Honestly Significant Difference *post hoc* test. Where statistically significant differences (*P* < 0.05) were found, compact letter displays were generated using the multcompLetters() function from the R package *multcompView* to assign groupings (67). Each strain was assigned a letter at each time point; strains sharing a letter are not significantly different from one another.

### RBP production and purification/recombinant protein production and purification

All predicted ORFs from P738 were subject to HHpred analysis. The putative receptor binding protein of P738 (protein id = QDP43720.1) was codon optimized for *E. coli* to improve protein expression using the “codon optimization” tool on VectorBuilder (https://en.vectorbuilder.com/tool/codon-optimization.html). The codon-optimized protein sequence was synthesized by Eurofins Genomics (Germany). The optimized RBP was amplified from the supplied vector using RBP primers listed in Table 2. The resulting amplicon was purified using a GenElute PCR cleanup kit (Sigma, USA) and cloned into the green fluorescent protein fusion vector pHTP9 (NZYTech, Portugal) following the manufacturer’s protocol. The ligation mixture was dialyzed against sterile distilled water before being introduced to *E. coli* BL21 (DE3) via the heat shock method (30 minutes on ice; 42°C for 45 seconds; 2 minutes on ice [65]). After recovery at 37°C for 1 hour with agitation (150 rpm), the cells were plated on LB agar containing 50 µg/mL kanamycin (Sigma, USA) and incubated overnight. Recombinant colonies were screened using pHTP9-specific primers (listed in Table 2) and verified by Sanger sequencing (Eurofins, Germany).

For protein production and purification, 100 mL of LB broth supplemented with 50 µg/mL kanamycin was inoculated with 1 mL of an overnight culture of *E. coli* BL21(DE3) harboring the GFP-P738 RBP fusion protein. Cells were grown to an OD_600_ of 0.5–0.6 before isopropyl-β-D-thiogalactoside (Melford Laboratories, UK) induction at a final concentration of 0.5 mM. The culture was incubated at 24°C for 24 hours with shaking (300 rpm). Cells were harvested by centrifugation at 4,700 × *g* for 30 minutes at 10°C. The resulting cell pellets were resuspended in lysis buffer (10 mM Tris-HCl [pH 7.5], 300 mM NaCl, 10 mM imidazole, and 25 mg/mL lysozyme) and stored at −70°C for a minimum of 24 hours. After thawing, the cells were subjected to five cycles of sonication (MSE Soniprep, Sanyo, Japan) at maximum amplitude (30 seconds on, 30 seconds off with a 2-minute rest on ice after the third cycle). Cellular debris was removed by centrifugation at 14,945 × *g* for 30 minutes at 4°C, followed by an additional centrifugation at 4,696 *× g* at room temperature to eliminate aggregates before protein purification.

The recombinant protein was purified using a standard Ni-nitrilotriacetic acid agarose column (Qiagen, UK), according to the manufacturer’s instructions, with the desired protein eluting in the 100–250 mM imidazole fractions. Protein concentrations were measured using the Qubit protein assay (Thermo Scientific, USA) on a Qubit 2.0 Fluorometer. Protein samples were dialyzed against protein buffer (10 mM Tris-HCl [pH 7.5] and 300 mM NaCl) before visualization via sodium dodecyl sulfate-polyacrylamide gel electrophoresis analysis. Protein fractions were stored at 4°C until use.

### Labeling/binding assays

Fluorescent binding assays were performed as previously described, with minor modifications (23). Briefly, 300 µL of relevant cells (Fig. 5) was harvested (6,000 × *g* for 5 minutes) at an OD_600_ between 0.4 and 0.6 and resuspended in 300 µL SM buffer before 30 µg of protein was added. The mixture was incubated at 42°C for 12.5 minutes. Cells were washed three times in 120 µL SM buffer before fluorescent binding was visualized by fluorescence microscopy. Fluorescence labeling of cells was visualized using an Olympus BX53 microscope equipped with an LED illuminator and fluorescence cube (U-FBNA), and images were processed using cellSens software (Olympus, Japan). An equal volume of SM buffer (representative of 30 µg) was added to cells acting as a negative control. *S. thermophilus* strain Brie28 (24) was selected as a non-host control.

### Preparation and structural analysis of RGP of *S. thermophilus* F1B

Approximately 8 L of *S. thermophilus* culture (UCCSt50 and F1B) was grown overnight and harvested by centrifugation at 4,400 × *g* at 4°C for 30 minutes. The cell pellets were washed twice with ice-cold sterile distilled H_2_O (300 mL, followed by pooling of cells before washing in 40 mL) and stored at −20°C.

CWPS (RGP) was extracted with cold 5% TCA and hot diluted HCl and purified on a Sephadex G-50 column, as described previously (20), without the final treatment with HF. HCl preparations had identical monosaccharide composition; the preparation of 0.01 M HCl extraction was used for linkage analysis. It was further purified by reverse-phase HPLC using an Agilent Zorbax C18 column (250 × 9 mm) in solvent A (0.1% TFA, vol/vol) and B (80% MeCN, vol/vol) at a flow rate of 3 mL/minute, starting from 3% B for 10 minutes then gradient to 100% B over 30 minutes. The UV detector was set at 220 nm, and fractions were collected every 1 minute. The CWPS (RGP) purified by HPLC was used for NMR analysis.

NMR experiments were carried out on a Bruker AVANCE III 600 MHz (^1^H) spectrometer with a 5 mm Z-gradient probe with acetone internal reference (2.225 ppm for ^1^H and 31.45 ppm for ^13^C) using standard pulse sequences cosygpprqf (gCOSY), mlevphpr (TOCSY, mixing time 120 ms), roesyphpr (ROESY, mixing time 500 ms), hsqcedetgp (HSQC), hsqcetgpml (HSQC-TOCSY, 100 ms TOCSY delay), and hmbcgplpndqf (HMBC, 70 ms long range transfer delay). Resolution was kept <3 Hz/pt in F2 in proton-proton correlations and <5 Hz/pt in F2 of H-C correlations. The spectra were processed and analyzed using the Bruker Topspin program. Monosaccharides were identified by COSY, TOCSY, and NOESY cross-peak patterns and ^13^C NMR chemical shifts. Aminogroup location was concluded from the high field signal position of aminated carbons (CH at 45–60 ppm). Connections between monosaccharides were determined from transglycosidic NOE and HMBC correlations.

Monosaccharide and methylation analyses were carried out as described previously (46).

## Data Availability

The wild-type strain UCCSt50 was resequenced as part of this study (previously deposited under the strain name *S. thermophilus* 4078, accession number CP065477 [23]). The genome sequences of *S. thermophilus* UCCSt50, F1B, and F2A have been deposited in GenBank under BioProject accession number PRJNA1267047, and their associated accession numbers are as follows: CP194174, CP194006, and CP194005.

## SUPPLEMENTAL MATERIAL

Supplemental tables aem.01238-25-s0001.docx Tables S1 to S4.

ASM does not own the copyrights to Supplemental Material that may be linked to, or accessed through, an article. The authors have granted ASM a non-exclusive, world-wide license to publish the Supplemental Material files. Please contact the corresponding author directly for reuse.
