# Supplementary material for: The cell surface-associated rhamnose-glucose polysaccharide represents the receptor of Streptococcus thermophilus bacteriophage P738
Source: Appl Environ Microbiol. 2025 Sep 12;91(10):e01238-25. doi: 10.1128/aem.01238-25 (PMC12542673; doi:10.1128/aem.01238-25)
Supplement: Supplemental tables — Tables S1 to S4. [file aem.01238-25-s0001.docx]

**Supplementary Material**

**Table S1:** Efficiencies of plaquing (EOP) of phage P738 on its *S. thermophilus* host UCCSt50 and panel of presumed BIMs.

| **Strain/BIM** | **EOP of P738** |
| --- | --- |
| UCCSt50 WT | 1 |
| F1A | ≤ 3 x 10^-8^ |
| F1B | 6.6 x 10^-7^ |
| F1C | ≤ 3 x 10^-8^ |
| F1D | 3.3 x 10^-7^ |
| F1E | ≤ 3 x 10^-8^ |
| F2A | 6.6 x 10^-7^ |
| F2B | ≤ 3 x 10^-8^ |
| F2C | ≤ 3 x 10^-8^ |
| F2D | ≤ 3 x 10^-8^ |
| F2E | ≤ 3 x 10^-8^ |
| F21A | ≤ 3 x 10^-8^ |
| F21B | ≤ 3 x 10^-8^ |
| F21C | ≤ 3 x 10^-8^ |
| F21D | 6.6 x 10^-7^ |
| F21E | 2.3 x 10^-5^ |
| F22A | ≤ 3 x 10^-8^ |
| F22B | ≤ 3 x 10^-8^ |
| F22C | ≤ 3 x 10^-8^ |
| F22D | ≤ 3 x 10^-8^ |
| F22E | 3.3 x 10^-7^ |

**Table S2:** General genome characteristics of UCCSt50 and selected BIMs, F1B and F2A. The quality or completeness of genome assemblies was evaluated with the microbial genomes atlas (MiGA) (61).

| **Strain** | **Genome size (bp)** | **Genome Completeness** | **Average GC content** | **No. of predicted proteins** | **Accession number** |
| --- | --- | --- | --- | --- | --- |
| UCCSt50 | 1841797 | 100 % | 39.1 | 1951 | CP194174 |
| F1B | 1839155 | 100 % | 39.1 | 1949 | CP194006 |
| F2A | 1839161 | 100 % | 39.1 | 1947 | CP194005 |

**Table S3:** CRISPR loci and spacer array profiles of *S. thermophilus* UCCSt50 and BIMs F1B and F2A

| **Strain** | **C1 (bp)** | **C1 (spacers)** | **C2 (bp)** | **C2 (spacers)** | **C3 (bp)** | **C3 (spacers)** |
| --- | --- | --- | --- | --- | --- | --- |
| UCCSt50 | 2,409 | 36 | 101 | 1 | 1,358 | 20 |
| F1B | 2,409 | 36 | 101 | 1 | 1,358 | 20 |
| F2A | 2,409 | 36 | 101 | 1 | 1,358 | 20 |

**Table S4:** NMR data for F1B polysaccharide (600 MHz, 40 °C, ppm).

| Sugar |  | H/C 1 | H/C 2 | H/C 3 | H/C 4 | H/C 5 | H/C 6 |
| --- | --- | --- | --- | --- | --- | --- | --- |
| α-Rha A | H | 5.07 | 4.27 | 3.96 | 3.57 | 3.80 | 1.32 |
|  | C | 100.8 | 77.6 | 79.5 | 72.8 | 71.1 | 17.9 |
| α-Glc B | H | 4.98 | 3.52 | 3.72 | 3.57 | 4.22 | 3.94; 3.94 |
|  | C | 99.2 | 72.7 | 74.2 | 70.3 | 71.5 | 67.4 |
| α-Rha C | H | 4.92 | 4.05 | 3.92 | 3.47 | 3.76 | 1.34 |
|  | C | 100.8 | 79.3 | 71.3 | 73.6 | 70.0 | 18.1 |
| β-Gal D | H | 4.64 | 3.55 | 3.67 | 3.93 | 3.68 | 3.75; 3.75 |
|  | C | 105.2 | 72.9 | 74.1 | 69.8 | 76.4 | 62.2 |
| α-Rha F | H | 4.87 | 3.81 | 3.74 | 3.43 | 3.99 | 1.24 |
|  | C | 102.5 | 71.9 | 71.5 | 73.2 | 70.1 | 17.7 |
| α-Rha F' | H | 4.87 | 3.82 | 3.96 | 3.69 | 4.06 | 1.32 |
|  | C | 102.5 | 71.9 | 71.5 | 82.3 | 68.7 | 17.9 |
| β-GlcNAc G | H | 4.74 | 3.74 | 3.64 | 3.47 | 3.47 | 3.75; 3.96 |
|  | C | 103.9 | 56.9 | 82.7 | 70.3 | 77.1 | 62.2 |
